# Is non-mentored initiation of laparoscopic colorectal surgery safe? Single surgeon initial experience with the first 40 cases

**DOI:** 10.3389/fsurg.2023.1196037

**Published:** 2023-09-05

**Authors:** Branko Bakula

**Affiliations:** Department of Surgery, University Hospital Sveti Duh, Zagreb, Croatia

**Keywords:** laparoscopy, colorectal resection, learning curve, colon cancer, laparoscopy—complications

## Abstract

**Introduction:**

Although laparoscopic colorectal surgery is now accepted as a standard procedure in treating colorectal cancer, the proportion of laparoscopically operated patients with colorectal cancer is still generally quite low. The aim of this study is to assess feasibility, safety, and outcomes of a non-mentored initiation of laparoscopic colorectal resections by a young surgeon without previous experience in laparoscopic colorectal surgery.

**Materials and methods:**

We analyzed the characteristics of the first 40 elective cases of laparoscopic colorectal resections performed by a single surgeon during the period between June 2019 and March 2022. All of the operations were performed without the attendance or supervision of an experienced surgeon in laparoscopic colorectal surgery. The patients were divided into three groups (the early, intermediate, and late group).

**Results:**

The conversion rate, complications rate, and postoperative recovery were similar among groups. The mean overall operative time was 219.5 min (range 130–420 min) and had reduced significantly during the learning curve (*p* = 0.047). The overall conversion rate was 12.5%. In two cases (5%), the oncological principles were violated (incomplete total mesorectal excision). In three patients (7.5%), intraoperative complications had occurred (small bowel injury, splenic injury, and significant bleeding from the minor peripancreatic artery). Three cases of major postoperative complications (Clavien–Dindo grade III) were recorded, two of which required reoperation (anastomotic bleeding and fascial dehiscence). There was no 90-day mortality reported. The overall mean number of lymph nodes retrieved was 12.45, which did not differ significantly among groups (*p* = 0.678). The average follow-up was 13.75 months (range 1–31 months). Cancer recurrence was recorded in four patients (10%). Port-site metastasis was not detected in any of the cases.

**Conclusion:**

A safe and non-mentored initiation of laparoscopic colorectal surgery with an acceptable rate of complications and acceptable oncological results can be achieved. Still, when compared with a structured initiation in a controlled environment with the supervision of an experienced surgeon in laparoscopic colorectal surgery, the results of a non-mentored initiation are worse in most of the fields, including operative time, conversion rate, complications rate, and duration of hospital stay. Therefore, I strongly recommend engaging young surgeons in fellowship programs on structured laparoscopic colorectal surgery whenever possible before starting performing these procedures on their own.

## Introduction

Since its first introduction in the early 1990s, the practice of laparoscopic surgery has spread very quickly and was easily adopted in hospitals worldwide. Due to the evident advantages of a minimally invasive surgery, such as less stress for the patient, faster recovery, less pain, shorter hospitalization, and better esthetic result, laparoscopic surgery has rapidly become the gold standard for treating numerous benign diseases, which is best recognized by treating cholecystolithiasis and acute appendicitis (1).

The introduction of laparoscopy had a much more difficult and longer path with regard to malignant diseases. The pioneers of laparoscopic colorectal surgery in their beginnings were greeted with considerable skepticism (2). In order for laparoscopic surgery for colorectal cancer to be implemented as a standard procedure, the procedure had to be justified by answering three basic questions: can all oncological principles be respected as in open surgery, whether the procedure is burdened with higher risk of complications compared with open surgery, and whether the procedure is even technically feasible with laparoscopic surgery.

Numerous large clinical randomized studies have been conducted to date that dealt with these issues, and it now becomes evident that laparoscopic colorectal surgery is a safe method that provides the same oncological outcomes as open surgery with all the advantages of minimally invasive surgery (3–5).

Although laparoscopic colorectal surgery is now accepted as a standard procedure in treating colorectal cancer, the proportion of laparoscopically operated patients with colorectal cancer is still very low outside of certain tertiary high-volume centers. Thus, recent studies have shown that the percentage of patients with colorectal cancer operated laparoscopically barely reaches 50% in the United States, while this percentage is certainly much lower for most of the rest of the world (6–9).

There are many reasons for this: a longer learning curve, a longer duration of the operation in the beginning with the consequent longer time occupying the operating room, lack of support from older colleagues, lack of an adequate education program, higher cost of the operation, and lack of adequate equipment (10, 11). Consequently, the development of laparoscopic colorectal surgery in to a particular center often rests on the enthusiasm of an individual who tries to start a program of laparoscopic colorectal surgery with great personal engagement.

The aim of this study is to assess feasibility, safety, and outcomes of a non-mentored initiation of laparoscopic colorectal surgery by a young surgeon without previous experience in laparoscopic colorectal surgery, as well as to analyze the learning curve during this process.

## Materials and methods

### Subjects

We analyzed the characteristics of the first 40 elective laparoscopic colorectal resections for colorectal cancer or unresectable colonic polyp performed by a single surgeon (BB). The operations were performed in the period between June 2019 and March 2022. Before starting the study, the surgeon had already undergone multiple educational programs on laparoscopic colorectal surgery through surgical workshops, educational online platforms, hands-on trainings, and observerships at high-volume centers (United States and Germany), but the cases in this study were his first independent cases of laparoscopic colorectal resections. Furthermore, the surgeon has performed approximately 60 open colorectal resections and already had significant experiences with laparoscopic surgery for benign diseases such as cholecystectomies and appendectomies. All of the operations in this study were performed without the attendance or supervision of an experienced surgeon in laparoscopic colorectal surgery.

When making a decision for a laparoscopic approach, the following were taken as contraindications: major or multiple previous laparotomies where problems with adhesions were expected, giant tumors infiltrating the surrounding organs, and morbid obesity.

## Methods

### Surgery and perioperative care

#### Preoperative preparation

All patients were admitted to the hospital a day before the schedule of the surgery, and this was also when the mechanical bowel preparation was performed using a non-absorbing osmotic agent (polyethylene glycol with ascorbic acid). During this preparation, the patients were on a liquid diet with the addition of two doses of an enteral nutrition drink.

#### Operative procedure

A prophylactic antibiotic therapy with cephalosporin and metronidazole was administered to all patients within half an hour after the first skin incision, and a second dose of antibiotics was administrated if the operation lasted longer than 4 h. Pneumoperitoneum was established using a Veress needle umbilically, maintaining an intra-abdominal pressure of 13–15 mmHg during the operation. The first trocar was placed umbilically, blindly, and a 0° camera was used. If adhesions around the umbilicus were expected, an open method of creating a pneumoperitoneum using the Hasson method was used. Depending on the type of operation, additional two to five working trocars were placed. The colon was mobilized using a medial-to-lateral approach in all cases.

All the resections were performed with a curative-intent treatment and by respecting the oncological principles of open radical surgery (12, 13):

1. Minimum proximal resection margin of 10 cm on colon;
2. Minimum distal resection margin of 10 cm on colon, 5 cm on the rectosigmoid and proximal third of the rectum, and 2 cm on middle and distal third of the rectum;
3. Adequate regional lymphadenectomy:
  a. Central ligation of the main feeding arteries (ligation of ileocolic artery at the origin of the superior mesenteric artery for right colon tumors, low ligation of the inferior mesenteric artery with preservation of left colic artery for sigmoid and rectal tumors, ligation of left colic artery and left branch of middle colic artery for tumors of the splenic flexure and descending colon);
  b. Partial or total mesorectal excision depending on tumor location.

All intestinal anastomoses were created with a circular mechanical stapling device. In right hemicolectomies, all ileocolonic anastomoses were created extracorporeal through a mini laparotomy (extraction site was the umbilicus or right rectal muscle) as termino-lateral ileocolonic anastomoses. In rectal resections, a small Pfannenstiel incision was used for extracting the specimen and placing of the anvil of a circular stapler. Colorectal anastomoses were created in a termino-terminal fashion under the laparoscopic control, and all of them were tested with an air test. In our institution, we only use protective ileostomy in selective cases of low anterior resections (LAR). The decision of creating protective ileostomy is being made intraoperatively for every patient individually, taking into account the present specific risk factors for anastomosis dehiscence. In all patients, a nasogastric tube was employed during the operation, and an abdominal drain was employed at the end of the operation.

#### Postoperative management

All patients were routinely admitted to the intensive care unit after the operation, and in the case of an uneventful early recovery, they were transferred to the ward on the first postoperative day. Postoperative recovery was carried out following the “enhanced recovery after surgery” protocol whenever possible. This included the early removal of the nasogastric tube and urinary catheter, early mobilization of the patient, early withdrawal of analgesia, stimulation of peristalsis, and early initiation of oral intake.

### Data assessed

All consecutive patients who underwent an elective laparoscopic colorectal resection by one surgeon (BB) were enrolled prospectively in a registry database recording the following groups of parameters: patient data (age, gender, tumor location), operative parameters (duration of surgery, intraoperative complications, dissection outside of the correct surgical-anatomical planes, oncological adequacy of the specimen, conversion to open surgery), early postoperative complications (using Clavien–Dindo classification), early postoperative recovery (first stool, initiation of oral intake, length of hospitalization), and long-term outcomes (oncological, surgery-related complications).

All operative procedures were video-recorded and subsequently analyzed in order to determine adequate operative data.

During the data analysis, the patients were divided into three groups according to the order of operation to evaluate the characteristics of the learning curve (Group 1—early group; Group 2—intermediate group; Group 3—late group).

### Statistical analysis

Data were analyzed using SPSS software (*IBM SPSS Statistics for Windows, Version 25.0; IBM Corp, Armonk, NY, USA*). Descriptive statistics was used to describe the basic features of the sample in the study (proportions for categorical data, mean and standard deviation for continuous variables, or median and interquartile range for variables that significantly deviated from the normal distribution). One-way ANOVA or non-parametric substitute Kruskal–Wallis test was used to determine if the three groups of patients differed significantly in measured outcomes.

Linear regression was used to determine the slope of the regression line and the trend in operative times of each additional case operated.

### Ethics

The study was approved by the Ethics Committee of Clinical Hospital Sveti Duh. All patients included in the study had given their informed consent prior to their inclusion. All procedures performed in studies involving human participants were in accordance with the ethical standards of the institutional research committee and with the 1964 Helsinki declaration and its later amendments or comparable ethical standards.

## Results

In the period between June 2019 and March 2022, 40 patients were submitted to elective laparoscopic colorectal resection for colorectal cancer or colonic polyp unfavorable for endoscopic excision performed by one surgeon (BB).

Of these patients, 19 were men and 21 were women. The average age of the patients was 68.65 years (range 44–81 years). In total, 13 right hemicolectomies, 13 high anterior resections (HAR), five low anterior resections, six left hemicolectomies, one abdominoperineal resection (APR), one total colectomy with ileorectal anastomosis (IRA), and one transverse resection were performed. A protective ileostomy was not needed in any of the patients (Table 1).

**Table 1:** Data/information on patients operated, according to patient groups and the total sample.

		Group						Total	
		Group 1 (1–13)		Group 2 (14–26)		Group 3 (27–40)		*n*	%
		*n*	%	*n*	%	*n*	%		
Operation	HAR	4	10.0%	4	10.0%	5	12.5%	13	32.5%
	Right colectomy	6	15.0%	3	7.5%	4	10.0%	13	32.5%
	Left colectomy	1	2.5%	2	5.0%	3	7.5%	6	15%
	LAR			4	10.0%	1	2.5%	5	12.5%
	Other	2	5.0%			1	2.5%	3	7.5%
Total		13	32.5%	13	32.5%	14	35.0%	40	100.0%

### Operative parameters

The average duration of the operation was 219.5 min (range 130–420 min). In five patients (12.5%), the procedure was converted to open surgery, while it was completed laparoscopically in the remaining 35 patients (87.5%). Three cases (7.5%) of intraoperative complications had occurred. Dissecting the wrong surgical layer was recorded in eight patients (20%), and the oncological principle of treatment was violated in two patients (5%).

**Figure 1:**
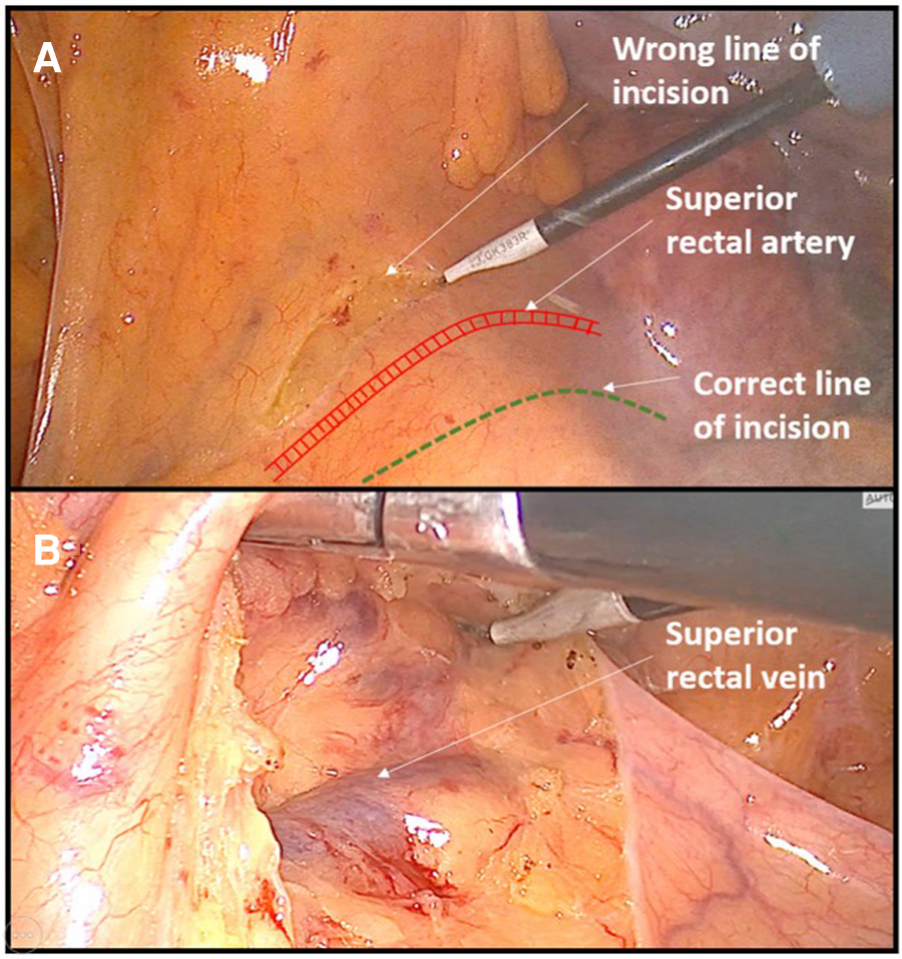
Example of dissecting the wrong surgical plane during anterior rectal resection. (**A**) Incision of peritoneum is performed above the area of superior rectal vessels instead of near the right iliac vessel as a landmark. (**B**) Consequently, dissection was performed out of the embryologic avascular plane above the superior rectal vessels.

**Figure 2:**
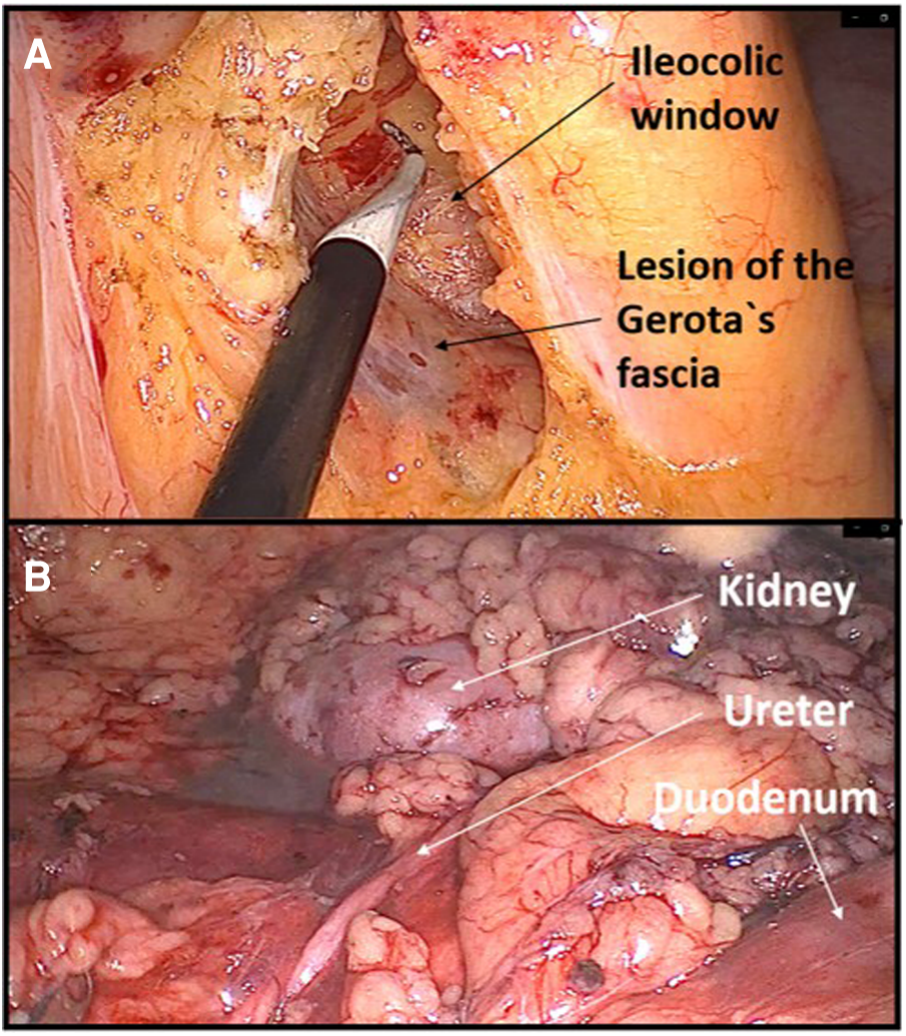
Example of dissecting the wrong plane during right hemicolectomy. (**A**) Lesion of Gerota's fascia with consequent dissection in the deep retroperitoneal space. (**B**) Dissecting through the perirenal fat exposing deep retroperitoneal structures.

**Figure 3:**
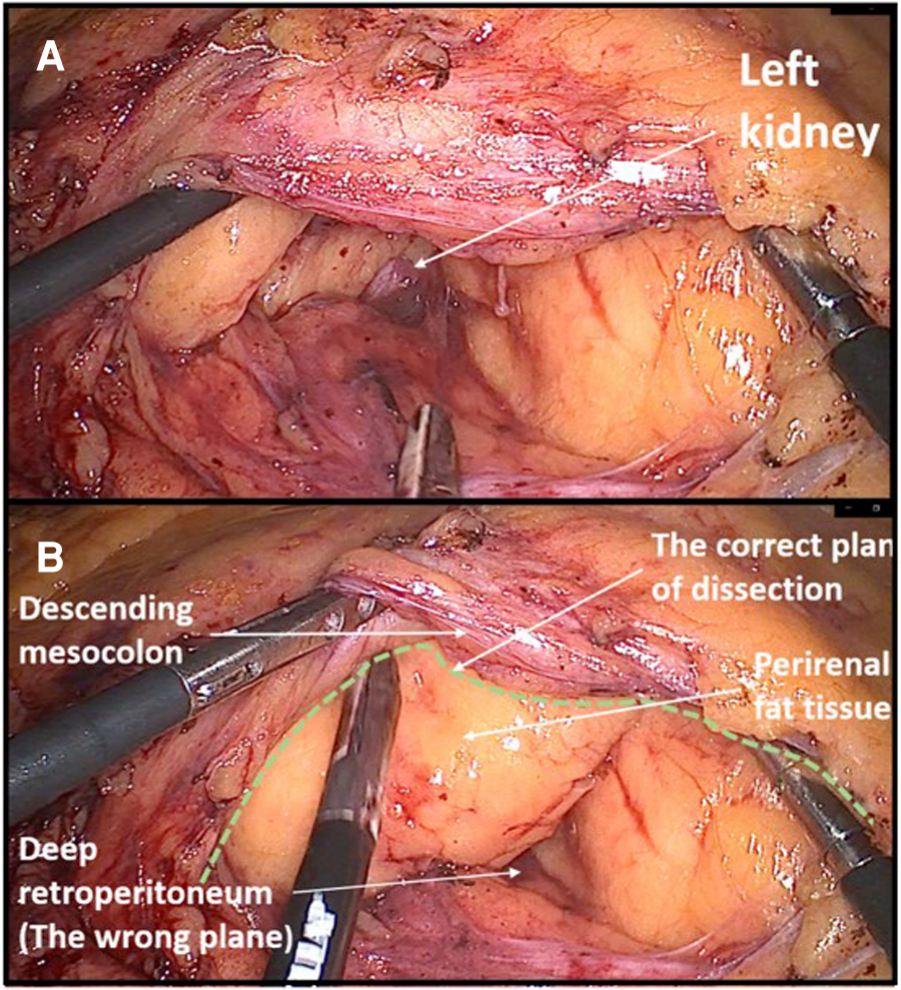
Example of dissecting the wrong surgical plane during left colectomy. (**A**) Due to a steep right tilt position of a patient and a surgical inexperience, the dissection was performed in the deep retroperitoneal plane through the perirenal fat exposing the lower pole of the left kidney. (**B**) Later, the correct avascular plane of dissection was found between the descending mesocolon and Gerota's fascia.

The ordinal number of procedures, type of procedures, and descriptions of incidents are described in Tables 2–5 and Figures 1–3.

**Table 2:** List of patients who were converted to an open procedure.

Ordinal number of a patient	Procedure	The reason for conversion
1	HAR	Obesity, technical difficulties
7	Left colectomy	Small bowel injury during adhesiolysis (previous umbilical hernia repair)
11	Right colectomy	Large (T4) cecal tumor infiltrating to the retroperitoneum
34	HAR	Obesity, technical difficulties
35	Left colectomy	Obesity, technical difficulties

**Table 3:** List of patients who experienced intraoperative complications.

Ordinal number of a patient	Procedure	Description of the complication and treatment method
7	Left colectomy	During laparoscopic part of left hemicolectomy, a small bowel injury had occurred while adhesions from prior open cholecystectomy were being dissected. Immediate conversion to open surgery and suture repair was done.
35	Left colectomy	During the open part of converted laparoscopic left hemicolectomy in an obese patient, a spleen injury had occurred, and splenectomy was performed. Blood loss was about 900 ml.
40	Left colectomy	During laparoscopic left hemicolectomy, arterial bleeding from the lower border of the pancreatic tail had occurred. Hemostasis was achieved by placing hemostatic sutures laparoscopically. Blood loss was about 550 ml.

**Table 4:** List of patients in whom dissecting the wrong surgical layers was recorded.

Ordinal number of a patient	Procedure	Description of a mistake
4	HAR	The initial peritoneal incision was done too high, above the level of inferior mesenteric vessels, resulting in a wrong dissection plane through the sigmoid mesocolon, which was accompanied with venous bleeding (Figure 1).
6	Right colectomy	Big part of dissecting was done through the ascending mesocolon instead of in embryologic plane between the ascending mesocolon and the Gerota's fascia.
9	APR	Mesorectal fascia injury, dissecting through the mesorectum.
14	LAR	Mesorectal fascia injury, dissecting through the mesorectum.
15	Left colectomy	During medial-to-lateral ascending mesocolon mobilization, instead of dissecting above pancreas entering the lesser sac, the dissection expanded deeply under the pancreas exposing the splenic vein.
18	Right colectomy	During medial-to-lateral right colon mobilization, the dissection plane was too deep, through the perirenal fat. The mistake was noticed when the lower pole of the left kidney was visualized. Too steep left lateral tilt of the patient was an important factor for the occurrence of this mistake (Figure 2).
24	Left colectomy	During medial-to-lateral splenic flexure mobilization, dissection was done under the Gerota's fascia, through the perirenal fat tissue. The mistake was noticed when the left kidney was visualized (Figure 3).
26	HAR	Dissecting under the left ureter during medial-to-lateral left colon mobilization.

**Table 5:** List of patients in whom violation of the oncological principles of treatment was detected.

Ordinal number of a patient	Procedure	Description of the mistake
9	APR	Damage to the mesorectal fascia with consequent retention of mesorectal fat tissue in the lower pelvis [“intramesorectal resection” according to Quirke et al. (14)].
14	LAR	Dissection from the inside of the mesorectal fascia in the lower half of the rectum with the consequent retention of mesorectal fat tissue in the pelvis [“intramesorectal resection” according to Quirke et al. (14)].

All patients had oncologic adequate margins of surgical resection. In all cases, an adequate regional lymphadenectomy was performed, which was determined by central ligation of the corresponding feeding blood vessels.

The average number of removed lymph nodes was 12.45. Since Levene's test of homogeneity of variances was not statistically significant, one-way ANOVA was calculated. No statistically significant differences were found among the three groups in the number of lymph nodes retrieved (*p* = 0.678) (Table 6).

**Table 6:** Comparison of lymph nodes retrieved and follow-up period among groups.

		Mean	SD	Median	Interquartile range		*N*	*p*
Lymph nodes	Group 1	12.46	4.235	11.00	10.00	15.00	13	*F*_(2,39) _= 0.393, *p* = 0.678
	Group 2	11.77	3.419	12.00	9.00	14.00	13	
	Group 3	13.07	3.751	12.00	10.50	18.00	14	
	Total	12.45	3.755	11.50	10.00	14.00	40	

### Postoperative complications

Early postoperative complications had occurred in nine patients (22.5%). According to the Clavien–Dindo grading system, three (7.5%) grade 1 complications, three (7.5%) grade 2 complications, and three (7.5%) grade 3 complications were recorded (Table 7). All complications were successfully treated conservatively except the bleeding from the ileocolonic anastomosis and dehiscence of the mini laparotomy wound after right hemicolectomy, which were treated by reoperation. The patient with ileocolonic anastomosis bleeding was revised on the same operative day when intraluminal arterial bleeding from the anastomosis was verified through a small colotomy, which was managed with a hemostatic suture. During the reoperation for dehiscence of the mini laparotomy wound, an intramuscular hematoma was found as the probable cause of the dehiscence.

**Table 7:** Comparison of the early postoperative complications according to groups.

Classification	Type of complication	Number of cases according to group		
		Group 1 (*N* = 13)	Group 2 (*N* = 13)	Group 3 (*N* = 14)
Clavien–Dindo I	Wound seroma	1	1	0
	Transient ileus	0	0	1
Clavien–Dindo II	Wound infection	1	0	0
	Paralytic ileus	0	1	1
Clavien–Dindo III	Anastomotic bleeding	1	0	0
	Fascial dehiscence	1	0	0
	Pulmonary embolism	0	1	0
Total number of complications by group		4/13 (30.76%)	3/13 (23.07%)	2/14 (14.28%)

There were no death cases reported within 90 days of the operation.

### Postoperative recovery

The average time until the first bowel movement was 2.68 days (range 1–7 days), the average time until starting a liquid diet was 3.5 days (range 2–8 days), while the average length of hospitalization was 9.98 days (range 7–20 days). When Levene's test of homogeneity of variances was statistically significant, Kruskal–Wallis test was used instead of ANOVA. No statistically significant differences were found among the three groups in early recovery indicators (*p*-values for starting liquid diet, the first stool, and the average length of hospitalization were 0.584, 0.730, and 0.789, respectively).

### Long-term results

The average follow-up period was 13.75 months (range 1–31 months). Cancer recurrence was recorded in four patients (10%). Port-site metastasis was not detected in any of the cases (Table 8).

**Table 8:** List of patients in whom recurrence of malignant disease was recorded.

Ordinal number of a patient	Procedure	Time from surgery (months) and method of detecting recurrence	Postoperative TNM stage; number and status of lymph nodes in specimen	Type of recurrence
4	HAR	12 months; surveillance colonoscopy	T4aN2bM0 (stage IIIC); Positive lymph nodes—(8/11)	Local anastomotic recurrence with metastases in the liver and regional lymph nodes
9	APR	13 months; surveillance MSCT	T3N1aM0 (stage IIIB); Positive lymph nodes—(1/21)	Distant solitary metastasis on the right lung measuring 2 cm without signs of local recurrence
12	Colon transversum resection	7 months; hospitalization due to deterioration of the general condition	T3N2bM0 (stage IIIC); Positive lymph nodes—(10/16)	Local anastomotic recurrence with malignant ascites, carcinosis of the peritoneum, and multiple metastases in the liver
18	Right colectomy	12 months; surveillance colonoscopy	T3N2aM0 (stage IIIB); Positive lymph nodes—(6/13)	Local anastomotic recurrence without signs of regional or distant metastases

MSCT, multislice computed tomography.

One case (2.5%) of late surgery-related complication was recorded. It was a case of a postoperative hernia in a patient who was reoperated on due to an early dehiscence of the mini laparotomy wound (right transrectal incision) after laparoscopic right hemicolectomy.

### The impact of the learning curve on intraoperative parameters

Analyzing the intraoperative data according to groups, progress was observed with regard to reducing the number of cases of dissecting the wrong surgical layers. Thus, four cases of dissecting the wrong layer were recorded in the first and second groups, while not a single case of dissecting the wrong surgical layer was recorded in the last group of patients (Table 9).

**Table 9:** Comparison of data on operations performed, according to groups of patients and the level of statistical significance of differences among groups.

		Mean	SD	Median	Interquartile range		*N*	*p* (ANOVA)	
Operative time	Group 1	237.69	72.47	220.00	185.00	255.00	13	*F*_(2,39) _= 3.314, *p* = 0.047	
	Group 2	235.38	50.93	220.00	202.50	262.50	13		
	Group 3	187.86	45.09	165.00	152.50	231.25	14		
	Total	219.50	60.381	212.50	175.00	243.75	40		
		Group						Total	
		Group 1 (1–13)		Group 2 (14–26)		Group 3 (27–40)		*n*	%
		*n*	%	*n*	%	*n*	%		
Intraoperative complications	No	12	30.0%	13	33.5%	12	30.0%	37	93.5%
	Yes	1	3.5%			2	5.0%	3	8.5%
Total		13	33%	13	33%	14	35%	40	100%
Dissecting in the wrong layer	No	10	25.0%	8	20.0%	14	35.0%	32	80.0%
	Yes	3	7.5%	5	12.5%			8	20.0%
Total		13	33%	13	33%	14	35%	40	100%
Conversion	No	10	25.0%	13	32.5%	12	30.0%	35	87.5%
	Yes	3	7.5%			2	5.0%	5	12.5%
Total		13	33%	13	33%	14	35%	40	100%
Oncologic principle violated	No	12	30.0%	12	30.0%	14	35.0%	38	95.0%
	Yes	1	2.5%	1	2.5%			2	5.0%
Total		13	33.5%	13	33.5%	14	35.0%	40	100%

Similar progress was observed with regard to reducing the average duration of the operation in the late group of patients. One-way ANOVA shows a statistically significant difference in operative times among groups. In the late group, the operative time is significantly shorter than that in the first two groups (*F*_(2,39) _= 3.314, *p* = 0.047) (Table 9). This will also be more adequately demonstrated during the analysis of the duration of the two most common types of surgery (right hemicolectomy and high anterior rectal resection) (Table 10).

**Table 10:** Average operative time of the two most common laparoscopic procedures according to groups.

Laparoscopic HAR							
	Mean	SD	Median	Interquartile range		*N*	*p* (Kruskal–Wallis)
Group 1	273.33	62.52	245.00	230.00	345.00	3	0.040
Group 2	211.25	25.62	217.50	185.00	231.25	4	
Group 3	173.75	38.21	162.50	148.75	210.00	4	
Total	214.65	55.29	220.00	165.00	235.00	11	
Laparoscopic right hemicolectomy							
	Mean	SD	Median	Interquartile range		*N*	*p*
Group 1	216.00	38.63	220.00	177.25	252.50	5	0.024
Group 2	211.67	7.64	210.00	205.00	220.00	3	
Group 3	151.25	17.02	155.00	133.75	165.00	4	
Total	193.33	40.02	192.50	165.00	220.00	12	

**Figure 4:**
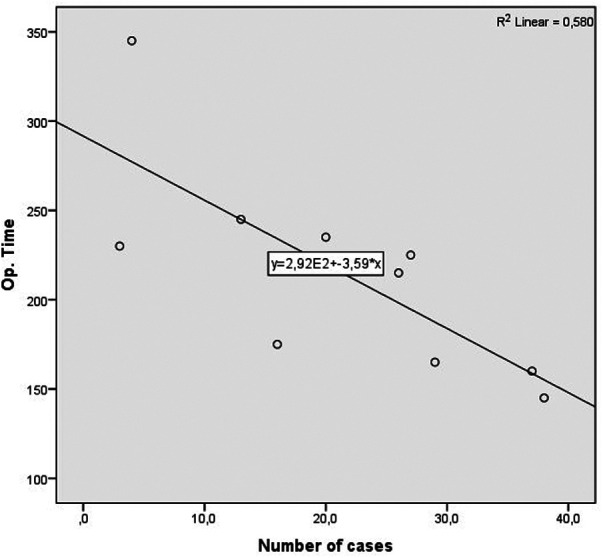
Operative time learning curve for laparoscopic HAR. A slope of −3.592 was determined, in the model that explained 53% of variance of operative time, and is statistically significant (*R*^2 ^= 0.580, Adj. *R*^2 ^= 0.533, *F* = 12.434, *p* = 0.008).

**Figure 5:**
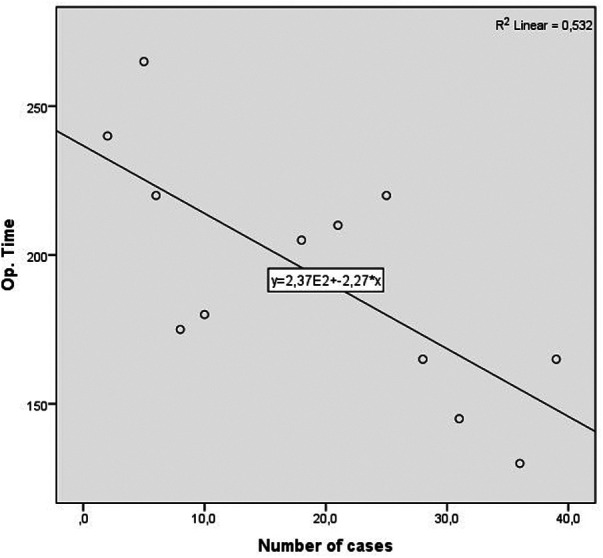
Operative time learning curve for laparoscopic right colectomy. A slope of −2.274 was determined, in the model that explained 58% of variance of operative time, and is statistically significant (*R*^2 ^= 0.532, Adj. *R*^2 ^= 0.485, *F* = 11.351, *p* = 0.007).

For an additional analysis of the learning curve, the operative time was analyzed separately for the two most common surgical procedures completed entirely with laparoscopic surgery (laparoscopic HAR and laparoscopic right hemicolectomy), when a statistically significant reduction in the duration of the surgical procedure was recorded in both cases, according to the groups determined by the order of operation (*p* = 0.040 and *p* = 0.024) (Table 10) (Figures 4, 5).

## Discussion

Safe laparoscopic colorectal surgery requires great knowledge and experience, while the technique itself is burdened by a longer learning curve compared with open resections. That is the reason why younger surgeons without adequate mentoring support from experienced colleagues find it difficult to start performing these procedures on their own. In more developed countries, there are specialized training programs for young surgeons for a specific area such as laparoscopic colorectal surgery. Such trainings are usually conducted in the form of official fellowships, which the surgeon attends in a highly specialized high-volume facility where he learns to operate under the mentorship of an experienced surgeon, and upon completion of the training, he returns to his home institution where he readily begins performing these operations independently. In Croatia, such a structured system of specialization has not yet been developed, which is why the possibility of learning laparoscopic colorectal surgery for a young surgeon depends on the institution where he completes his abdominal surgery residency and on the willingness of competent surgeons to educate him.

A study by Kim et al. described an example of the optimal initiation of laparoscopic colorectal surgery for a young surgeon (15). In this study, the results of the first 143 laparoscopic colorectal resections of a young surgical fellow in a high-volume hospital in South Korea were analyzed. The first 70 operations were performed under the supervision of a very experienced laparoscopic surgeon (who had performed more than 700 laparoscopic colon resections), while the last 73 operations were performed without supervision. It should be emphasized that before starting independent colorectal resections, the fellow underwent a very structured part of surgical training: performed at least 90 basic laparoscopic procedures, assisted more than 50 laparoscopic colorectal resections, reviewed more than 100 h of educational videos on laparoscopic colorectal resection, and trained more than 30 h in a laparoscopic simulation lab. This is probably the main reason for the excellent results published in this study. Thus, there was no statistically significant difference between the early and late groups in the average duration of the operation (220 vs. 222 min for colon cancer and 262 vs. 292 min for rectal cancer), the average blood loss (58 ml vs. 48 ml), or the number of intraoperative complications (5 vs. 6). During the entire study, only one case of conversion to an open procedure was recorded, and that case was in the early group due to ureteral injury. The overall morbidity was similar in both groups (27.1% and 26%). In all patients, oncological principles were respected, and a slightly higher average number of removed lymph nodes were recorded in the late group (31.7 vs. 23.8). No surgery-related mortality was reported. The rate of anastomotic leakage in rectal resections was slightly higher in the early group (12.8% vs. 8.1%). A potential cause of this may be using higher average number of stapler firings for cutting the rectum in the early group (2.46 vs. 1.97). A significant difference in the rate of use of three or more stapler firings between the early and late groups (38.5% vs. 9.8%) was also detected.

Another example of a well-structured education and controlled initiation of laparoscopic colorectal resections can be seen in the work of Luglio et al. (16). They presented the results of the first 50 operations of a young surgeon supervised by a senior experienced surgeon in open colorectal surgery. Before starting operating alone, the young surgeon completed an observership and fellowship programs in highly specialized colorectal units in the United States and the United Kingdom, where he observed and assisted in operations. Taking into account the division of subjects into the first 25 operations and the last 25 operations, the average operation time for the two most common operations, high anterior resection and right hemicolectomy, decreased from 251 to 187 min and from 200 to 147 min, respectively. Oncological principles were respected in all patients. The overall morbidity was 24%, while only two patients (4%) suffered from severe complications (Clavien–Dindo III). One case of bleeding from the anastomosis was reported, which was treated endoscopically, and one case of pelvic hematoma was also recorded.

Heroor et al. concluded in a 2015 study that a surgeon with extensive experience in open laparoscopic surgery needs about 30 cases with laparoscopic colorectal resections to reach the plateau of the learning curve (17). In this study, the results of the first 101 cases of one surgeon were presented, and it was shown that the average duration of the operation and intraoperative blood loss were reduced after the 30th operation, 182 vs. 162 min and 111 vs. 81 ml. The length of hospitalization in the late group was also shorter, which was 8 days compared with 11.5 days. There were no statistically significant differences in the number of removed lymph nodes among the first 30 cases and the last 70 cases. As for the severe early complications, one case of intra-abdominal abscess, three cases of anastomotic leakage, and one case of major dehiscence of the low colorectal anastomosis, which ended in the death of the patient, were recorded. Late complications included one rectovaginal fistula and two fecal fistulas.

In a retrospective study, Teixeira et al. (18) divided their first 43 cases of laparoscopic colorectal resection into three groups according to the order of operation, and they observed a shortening of the operative time (246 vs. 225 vs. 217 min), a decrease in the number of conversions to an open procedure (3 vs. 1 vs. 1), and an increase in the number of patients operated on for cancer (2 vs. 6 vs. 6). Three serious complications that required reoperation had occurred: anastomotic bleeding, small bowel obstruction with segmental small bowel necrosis, and necrosis of mobilized colon.

My beginning with laparoscopic colorectal surgery was an example of a non-mentored independent initiation of this method in an institution where laparoscopic colorectal resections were performed only sporadically. The decision to start this new method was of course not an easy one. In addition to doubts about the technical feasibility and support of the institution, the issue of ethics and the possible threat to the patients as potential victims of my own learning curve existed. Many years of preparation with regard to a large number of open colorectal resections, coping with serious complications of colorectal surgery, extensive experience with laparoscopic operations for benign diseases (cholecystectomies, appendectomies), attending numerous educational programs on colorectal resections in the form of observerships in international high-volume centers (USA, Germany), and hands-on courses were key for my own maturation to the point where I was completely convinced of my own readiness to start laparoscopic colorectal surgery on my own.

During the initiation, I insisted on operating slowly in the correct surgical and embryological layers with a low decision threshold for conversion to an open procedure in case of any ambiguity.

A large part of the operational data for this study was obtained by analyzing the video of each individual operation with the aim of detecting potential mistakes as objectively as possible. Also, it should be emphasized that the analysis of recordings of my own operations helped me a lot in improving the operating technique due to the easier possibility of noticing minor or major omissions that I was not aware of during the operation itself.

The most obvious progress detected during the analysis of intraoperative parameters was the shortening of the operative time as well as the absence of cases with dissecting the wrong surgical layer in the late phase.

Almost all cases of dissecting the wrong surgical layers were the result of inexperience. In anterior rectal resections, due to the beginner's fear of injuring the large blood vessels (aorta and iliac vessels), the primary incision on the peritoneum was created too high (instead of near the right iliac artery as a landmark), which resulted in dissecting the wrong layer, most often above the superior rectal vessels. Any such dissection outside the avascular embryological layers was accompanied by frequent minor bleeding, which resulted in a significant prolongation of the operative time.

Another beginner's mistake that I would like to emphasize is not adjusting the visual perception of the operating field during the change in the position of the abdominal organs due to a more extreme tilting of the patient on the operating table. The patient's left tilt during right hemicolectomy and right tilt during left hemicolectomy are considered the standard during the laparoscopic approach because they greatly facilitate primary access to the root of the ileocolic vessels or inferior mesenteric vein. Danger threatens if the operator does not perceive in his own mind that the patient is tilted, which can lead to incorrectly dissecting the deep retroperitoneal layers. During my own learning curve, this happened to me twice, once during a right hemicolectomy and once during a left hemicolectomy where I dissected deep below the Gerota's fascia in both cases. I only became aware of the mistake when I noticed the kidney. Such instances of dissecting the wrong layers are primarily a consequence of inexperience, but to a lesser extent also one specific characteristic of laparoscopic surgery, which is pneumodissection. Pneumodissection is one of the most prominent advantages of laparoscopic surgery compared with open surgery, but it can also be misleading for a beginner because even wrong layers can be made to appear to be the right ones.

The conversion rate is a good indicator of the level of progress during the acquisition of a particular laparoscopic skill. The overall conversion rate in my initial experience was 12.5%. If we analyze the causes of conversions, we see that in three out of five cases, the cause of the conversion was “technical difficulties,” and it was due to the obesity in all three cases. The appearance of two of these conversions in the late group clearly indicates that the learning curve in my case has not ended yet. With an experienced laparoscopic surgeon, obesity is rarely a reason for conversion, and I am confident that a more experienced surgeon would have easily completed these cases laparoscopically. This is a field in which I see a lot of place for improvement, primarily with regard to wiser positioning of trocars, use of a 30 degree laparoscope, and better use of assistant working trocars. All that together would result in a better exposure of the surgical field.

The basic part of any serious analysis of the effectiveness of treating cancer patients is following the principles of oncological resection during surgery, as well as long-term oncological results. In treating colorectal cancer, there are clear oncological criteria that must be respected. By analyzing my first 40 patients, we can see that the intraoperative oncological principles with regard to the length of the resection margins and the central ligation of the associated blood vessels were respected in all patients. A violation of oncological principles was observed in two patients with rectal cancer in whom there was partial damage to the mesorectal fascia (intramesorectal resection according to Quirke et al.) (14). The first patient had cancer of the middle third of the rectum, in whom low anterior resection was performed. The postoperative stage of the disease was T3N1M0 (stage IIIB), and no signs of recurrence were reported after 2 years. In the second case, the patient had locally advanced carcinoma of the distal third of the rectum who had undergone neoadjuvant chemoradiotherapy following abdominoperineal resection of the rectum. The postoperative pathological examination indicated a disease stage of T3N2M0 (stage IIIB). A PET-CT scan conducted 2 years and 6 months after surgery showed a nodule in the lower part of the lung with a diameter of 2 cm, which was suspected of metastasis. His tumor markers appeared normal. To what extent this suspected distant recurrence in this patient is the result of an imperfect total mesorectal excision or an already advanced disease at the time of surgery is difficult to estimate. Damage to the mesorectal fascia during total mesorectal excision is a common problem even among more experienced surgeons. Nevertheless, it is very important that a young surgeon strive for perfection during each operation when performing rectal surgery, which will eventually lead to minimizing the rate of imperfect specimens and consequently to reducing the risk of disease recurrence.

The results of an early postoperative recovery were in line with today's knowledge of faster recovery in laparoscopically operated patients. A significantly shorter time to the first stool, an earlier start of peroral feeding, and a shorter hospitalization were observed in patients operated completely with laparoscopic surgery compared with five converted patients. Postoperative paralytic ileus was recorded in only two patients, which was successfully treated conservatively. Two early postoperative complications had occurred that required a reoperation. The first, dehiscence of the mini laparotomy wound in the area of the right rectus as a result of intramuscular hematoma, was easily treated in reoperation. The second complication was life-threatening. There was severe bleeding from the termino-lateral ileocolonic anastomosis created with a mechanical circular stapler. The patient suffered from profuse rectorrhagia and was reoperated on the same evening in a state of a hemorrhagic shock when a pulsatile bleeding from a smaller artery on the anastomosis was visualized through the small colotomy. One hemostatic suture was successfully placed, and the patient experienced a smooth recovery after the operation. In our institution, almost all ileocolic anastomoses after right hemicolectomy are performed following this procedure, and this is the first time we have had this type of complication. The exact cause of the bleeding is not entirely clear. Malfunction of stapler, insufficient cleaning of the ileum from the mesentery during the placement of the purse string instrument, epiploic that entered the stapler line, high blood pressure, coagulation disorder, and administering excessive intravenous fluid during and after surgery are some of the possible causes. In any case, this complication has taught us to always check the ileocolic anastomosis created in this way by a direct visualization through the colon before it is closed.

With regard to the analysis of long-term oncological results, it should be emphasized that due to the relatively short average follow-up time (12 months, range 2–31 months), we cannot fully objectively judge the oncological results in this group. However, taking into account the number of recurrences (4/40), the TNM stage of the disease at the time of surgery in patients who had a recurrence (stage IIIB and IIIC), and the follow-up time, I believe that the recurrence rate is in the expected range.

The opinion with regard to the adequate number of cases to achieve proficiency in laparoscopic colorectal surgery varies a lot among surgeons as well as among studies. Although, we encounter only 30 or so cases, unfortunately, it is more possible that the real numbers are much higher. Thus, we highlight the results of one valuable systematic review conducted by Miskovic et al. (19) who, based on the analysis of seven large international studies that included 19 surgeons and 4,852 cases, concluded that the length of the learning curve in laparoscopic colorectal surgery is: 152 cases for conversion, 143 cases for complications, 96 cases for operating time, 87 cases for blood loss, and 103 cases for length of hospitalization. They also observed that high body mass index and rectal cancer, especially in men, are independent risk factors for complications and conversion, which was also proven in my experience.

After performing the first 40 independent laparoscopic colorectal resections, I am sure that the learning curve in my case has not ended yet. This especially applies to technically more difficult surgical procedures, such as procedures involving the mobilization of the splenic flexure and low rectal cancers that require dissecting deep into the pelvis. Personally, I am of the opinion that the learning curve for a specific surgical procedure is not completed only at the moment when objective, numerically measurable operating parameters show it, but when the surgeon himself feels safe, confident, and relaxed before entering the operating room. The moment when the surgeon, upon entering the operating room, feels that the patient is safe in his hands and that he is providing him with the best possible care, and his results so far support this, is the best indicator of the end of the learning curve.

Considering that for minor laparoscopic surgical procedures such as cholecystectomy or appendectomy, I developed this sort of confidence only after operating on approximately 100–150 patients; it is logical to expect that I will need at least the same number of procedures for laparoscopic colorectal resections.

A non-mentored initiation of laparoscopic colorectal surgery can be challenging and also dangerous. Although the complications rate and oncological results in my case are within the acceptable range, nevertheless, comparing my results with the results of similar studies of learning curve analysis in a young surgeon but supervised by an experienced surgeon in laparoscopic colorectal surgery, worse results are clearly observed in my case, which indicates that controlled initiation is much safer (20–24). That is why I am of the opinion that surgeons in countries where an educational platform for laparoscopic colorectal surgery is not available or is not well organized should not easily engage in these procedures on their own. But if they decide to perform the surgery alone, the following preconditions for a successful procedure are mandatory:

• strong motivation and great effort invested in education, which includes the obtaining of theoretical knowledge as well as practical skills through observerships, surgical workshops, and hands-on training courses;
• great experience with open colorectal resections;
• great experience with laparoscopic surgery for benign diseases (cholecystectomies, appendectomies);
• support of older experienced surgeons in open colorectal surgery in their home institution;
• support of colleague surgeons and intensive care specialists with experience in dealing with complications specific for colorectal surgery;
• adequate equipment for performing laparoscopic procedure; and
• responsible approach and psychological preparation for each individual case with careful and slow operating technique accompanied by a low threshold for converting to open surgery in case of any difficulties or dilemmas.

## Conclusion

Although the laparoscopic approach to colorectal resections is currently considered the standard, its general acceptance worldwide is not at the expected level. The reason for this is, on the one hand, a longer learning curve and, on the other hand, a lack of institutional support and adequate education.

A safe and non-mentored initiation of laparoscopic colorectal surgery with an acceptable rate of complications and acceptable oncological results can be achieved. Still, when compared with structured initiation in controlled environment with the supervision of an experienced surgeon in laparoscopic colorectal surgery, results are worse in most of the fields including operative time, conversion rate, complications rate, and duration of hospital stay. Therefore, I strongly recommend engaging young surgeons in fellowship programs on structured laparoscopic colorectal surgery whenever possible before starting performing these procedures on their own.

## Data Availability

The original contributions presented in the study are included in the article/supplementary materials, further inquiries can be directed to the corresponding author.
